# Tailoring the Mechanical Properties of Fungal Mycelium Mats with Material Extrusion Additive Manufacturing of PHBH and PLA Biopolymers

**DOI:** 10.1021/acsomega.4c07661

**Published:** 2024-12-03

**Authors:** Huaiyou Chen, Sophie Klemm, Antonia G. Dönitz, Yating Ou, Bertram Schmidt, Claudia Fleck, Ulla Simon, Christina Völlmecke

**Affiliations:** † Faculty III Process Sciences, Institute of Materials Science and Technology, Chair of Advanced Ceramic Materials, Technische Universität Berlin, Berlin 10623, Germany; ‡ Faculty III - Process Sciences, Institute of Materials Science and Technology, Chair of Materials Science & Engineering/Fachgebiet Werkstofftechnik, Technische Universität Berlin, Str. des 17. Juni 135, Berlin 10623, Germany; § Faculty V Mechanical Engineering and Transport Systems, Institute of Mechanics, Chair of Stability and Failure of Functionally Optimized Structures, Technische Universität Berlin, Berlin 10623, Germany; ∥ Faculty III Process Sciences, Institute of Biotechnology, Chair of Applied and Molecular Microbiology, Technische Universität Berlin, Berlin 10623, Germany

## Abstract

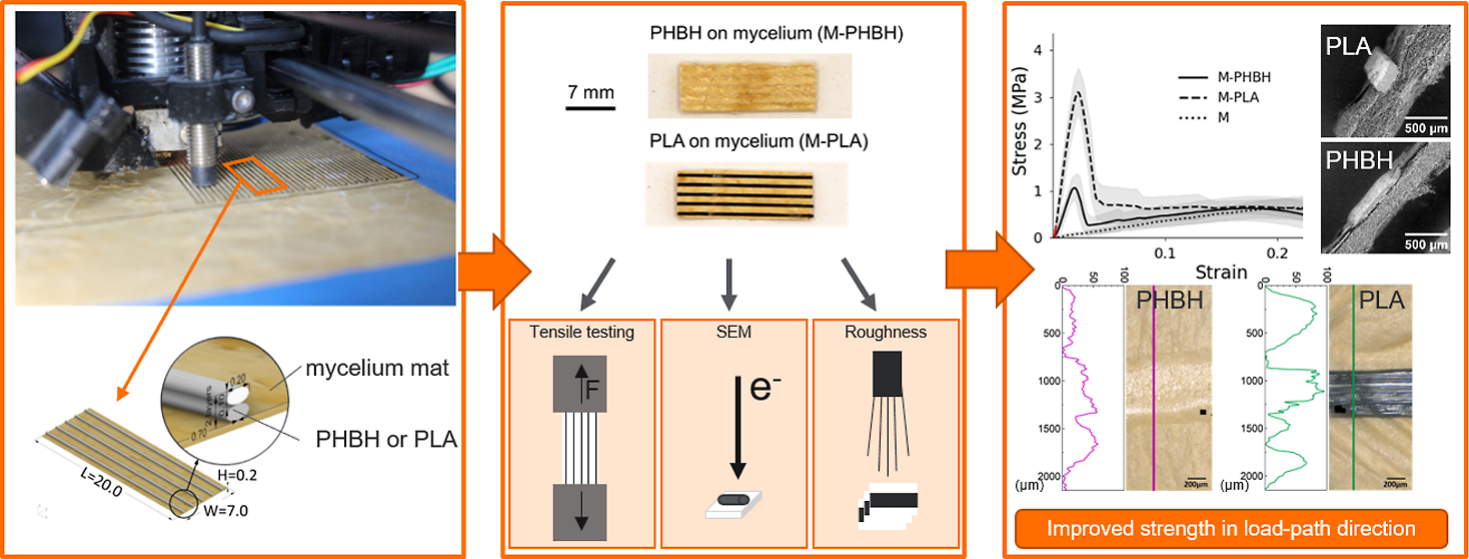

To advance the concept of a circular economy, fungal mycelium-based materials are drawing increased attention as substitutes for nonsustainable materials, such as petroleum-based and animal-derived products, due to their biodegradability, low carbon footprint, and cruelty-free nature. Addressing the challenge of mechanical properties in fungal mycelium products, this study presents a straightforward approach for reinforcing fungal mycelium mats. This is achieved by using two bio-based and biodegradable polymers, poly(3-hydroxybutyrate-*co*-3-hydroxyhexanoate) (PHBH) and polylactic acid (PLA), via material extrusion additive manufacturing (MEX AM), commonly known as 3D printing, to produce fungal mycelium-biopolymer composites. By analyzing the mechanical properties, roughness, and morphology, this study demonstrates significant improvements in ultimate tensile strength with the application of PHBH and even more with PLA, while elasticity is reduced. The study also discusses potential improvements to enhance the quality of the fungal mycelium-biopolymer composites without trading off bio-based and biodegradable features, offering a promising pathway for the development of more durable and sustainable fungal mycelium products.

## 1 Introduction

From a sustainable perspective, further renewable bio-based and biodegradable materials must urgently be developed to replace unsustainable, nonbiodegradable, and/or hazardous synthetic materials on the market, such as petroleum-based materials.^1,2^ The focus lies on efficient resource utilization without competing with food cultivation and supply. Fungal mycelium-based materials are an emerging class of these materials,^3−8^ usually made from cultures of filamentous fungi such as basidiomycetes *Fomes fomentarius* and *Ganoderma lucidum* (classified as biosafety level 1^9−12^). The use of *F. fomentarius* as a material source can be traced back to Stone Age,^13^ and the species is also used in traditional medicines^14,15^ and clothing.^16^ Fungal mycelium is the vegetative part of a fungus that mainly consists of glucan, chitin, protein, and mannan^17^ and is usually dried or deactivated when used as fungal mycelium-based materials. Compared to plants that also serve as a source of renewable and biodegradable material, fungal mycelium has shorter growth cycles, requires less water, and can be grown in vertical farms without sunlight.^18,19^ As they consume plant-derived biomass during growth, for example, the lignocellulosic remnants from agriculture, forestry, or the food sector, they are suggested for increasing resource efficiency through the upcycling of unused or low-value biomass. This can be a significant driver in the transition to a circular economy.^3^ Fungal-based materials are considered or are already on the market for substituting non-load bearing components in the construction sector, such as thermal and acoustic insulation panels^20−23^ and self-healing concrete.^24,25^ They can also be used for the synthesis of chitin or chitosan-based materials,^26,27^ packaging materials,^28,29^ animal-free leather,^30−33^ meat substitutes,^34,35^ or even biomedical scaffolds^36^ in the form of biocomposites or pure mycelium mats. Pure fungal mycelium mats can be produced either by solid-state surface fermentation (fungal growth on solid biomass), or liquid-state surface fermentation (fungal growth on liquid culture) as well as stirred submerged liquid fermentation (fungal growth in liquid culture).^30^ Due to the inherent nature of fungal mycelium, the impact of improving the characteristics, such as tensile strength, of the product through fermentation conditions is limited so far, and post-processing is inevitable for many applications. However, not much is reported in the literature about characteristics and post-processing. Common methods of altering the mechanical properties of mycelium include chemical treatments (e.g., glycerol^37^ and corn-zein^38^) to increase elasticity and avoid brittleness as well as heat or cold pressing to increase tensile strength.^39,40^

Material extrusion additive manufacturing (henceforth called MEX AM),^41^ also known as 3D printing, on a substrate has already been used in many different areas to combine the flexibility of the substrates with the mechanical strength and potentially electronic or other functional properties imparted by the printed material. The additive manufacturing process of polymers on textiles^42^ or paper^43^ and other substrates^44^ leads to surprising material combinations with exciting mechanical properties and offers freedom and potential of tuning different material properties.^45^ Mycelium in combination with 3D printing has already been explored in the extrusion of the mycelium itself.^46−48^ Architectural structures can be built using mycelium-covered 3D-printed polymer building blocks.^49^

Polylactic acid (PLA) is one of the most used bio-based and biodegradable thermoplastic polyesters whose monomer lactic acid is produced by the fermentation of carbohydrates such as food wastes, corn stover, and sugar beets.^50−52^ PLA is superior to many petroleum-based polymers from a sustainability point of view, in addition to having good strength.^53,54^ It is also often combined with other biomaterials to form biocomposites to improve its toughness and thermal properties.^55,56^ PLA is compostable but only under certain industrial composting conditions.^57^ An alternative is poly(3-hydroxybutyrate-*co*-3-hydroxyhexanoate) (PHBHHx or PHBH), which is a type of polyhydroxyalkanoates that can also be produced by microorganisms and used as an alternative to petroleum-based materials.^58^ It can be adapted into conventional plastic industry processing systems but with biodegradability under aerobic, anaerobic, or marine conditions.^59^

In this approach, we propose a novel, yet simple, approach for enhancing and tailoring the mechanical characteristics of fungal mycelium mats from liquid-state surface fermentation by depositing defined patterns of different biopolymers on them using MEX AM. Herewith, we demonstrate a novel approach that will build the foundation for innovative and sustainable load-path-optimized biocomposites for various applications (e.g., flexible devices, interior design, etc.) while guaranteeing resource efficiency.

## 2 Results

### 2.1 Stress–Strain Analysis of Tensile Samples

**Figure 1:**
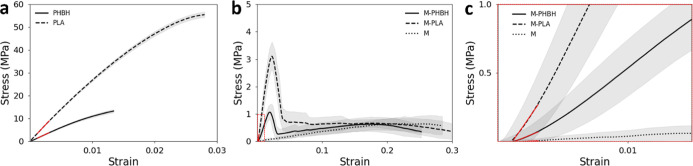
Stress–strain curves from tensile tests on (a) pure PHBH and PLA specimens produced by additive manufacturing, (b,c) fungal mycelium mats without or with PHBH and PLA printed on them. In (c), the initial part of the curves is shown in a close-up (marked red in b). The red tangents mark the initial part of the curve used for calculating the Young’s modulus.

In Figure 1a, the results of the uniaxial tensile test of specimens produced from pure material extrusion additive manufactured biopolymers PHBH and PLA are presented in the mean curves of the stress versus strain behavior with a 95% confidence interval. PLA exhibits a higher tensile strength (∼56 MPa) and Young’s modulus (∼2.9 GPa) compared to PHBH (∼13 MPa and ∼1.3 GPa, respectively). The stress–strain curves of PHBH and PLA show an initial steep slope; after that, the curve slope decreases and finally a brittle fracture occurs. In contrast, the biopolymer-mycelium composites depicted in Figure 1b show an initial increase and a peak where after the stress drops, the stress response following the peak is similar to pure mycelium (also shown in Figure 1b). Thus, the combination of the materials (M-PHBH and M-PLA) leads to an increase in tensile strength and Young’s modulus (see Table 1). The fungal mycelium mat (M) shows a very low tensile strength (∼0.65 MPa), a low stiffness (∼4.5 MPa), and a long elongation until fracture.

**Table 1:** Young’s Modulus and Ultimate Tensile Strength of Pure PHBH, Pure PLA, and the Fungal Mycelium Mats with and without Printed PHBH and PLA

	Young’s modulus/MPa	ultimate tensile strength/MPa
PHBH	1267.1 ± 44.3	13.31 ± 0.7
PLA	2859.6 ± 57.1	55.5 ± 1.2
M	4.5 ± 1.7	0.65 ± 0.2
M-PHBH	38.6 ± 19.65	1.1 ± 0.3
M-PLA	136.0 ± 57.7	3.11 ± 0.5

### 2.2 Prediction of the Effective Young’s Modulus of the Fungal Mycelium-Biopolymer Laminate Using the Voigt Model

We apply the rule of mixtures presented in the Materials and Methods sections on the stiffness results derived from the experimental investigations (PHBH, PLA, and M) and the mean cross-section area (*A*). This results in a Young’s modulus of 68 MPa for PHBH printed on the mycelium mat (Table 2), exceeding the experimental value by a factor of 1.8 times (Table 1). For PLA printed on the mycelium mat, the theoretical Young’s modulus is 177 MPa, which accounts for a factor of 1.3 of the experimental value.

**Table 2:** Analytically Derived Effective Young’s Moduli (*E*_eff_) of the Fungal Mycelium Mats Printed with PHBH or PLA^a^

	equivalent printed fiber	M-PHBH	M-PLA
*A*/mm^2^	0.16	3.12	2.60
*E*_eff_/MPa	/	68	177

a *A* stands for the mean cross-section area.

### 2.3 Morphology

**Figure 2:**
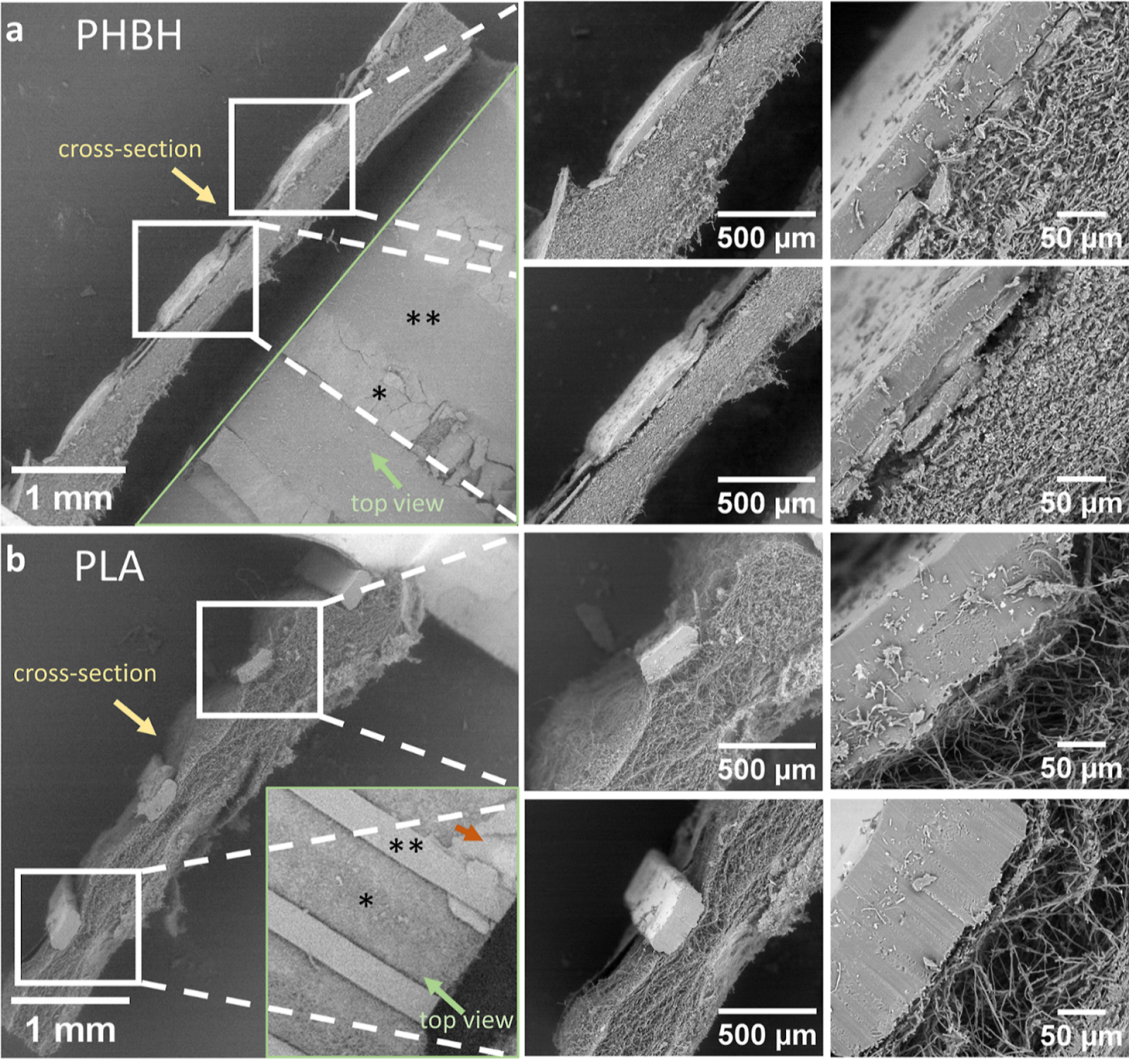
SEM images of (a) PHBH and (b) PLA printed on mycelium mats. The cross-section of two printed lines each is shown at different magnifications. The top view is shown in the same image for PHBH and an inlet is inserted for PLA. For clarification, the mycelium between the polymer lines is marked with a star, and the polymer lines are marked with two stars. The orange arrow indicates an area of an additional mycelium layer. The magnifications used are ×25, ×100, and ×500 from left to right.

Figure 2 shows the scanning electron microscopy (SEM) images of PHBH and PLA printed on fungal mycelium mats. For both variations, the top view and the cross-section can be seen on the left. For the cross-section, higher magnifications are depicted on the right. The PHBH printed on mycelium shows broad lines. At the highest magnification, you can see that there is another layer between the mycelium and the PHBH. This layer is under the PHBH, and there is good adhesion of the PHBH to this layer, while it seems to peel off from the mycelium. The star marks the mycelium between the PHBH lines. Here, cracks in the unexpected additional layer can be observed. The PLA printed on mycelium has remained more in its intended form (Figure 4b). Here, only a very thin additional layer is visible and the polymer is in direct contact with the loose hyphae of the mycelium. The orange arrow shows that a dense layer may also be present on M-PLA samples. To show that the unexpected dense layer of mycelium on top of the fungal mycelium mat is not from the MEX AM process, Figure S1 shows the fungal mycelium mat without polymer lines. It exhibits a dense, cracked layer of mycelium on top of a compressed, but rather loose, hyphal network.

**Figure 3:**
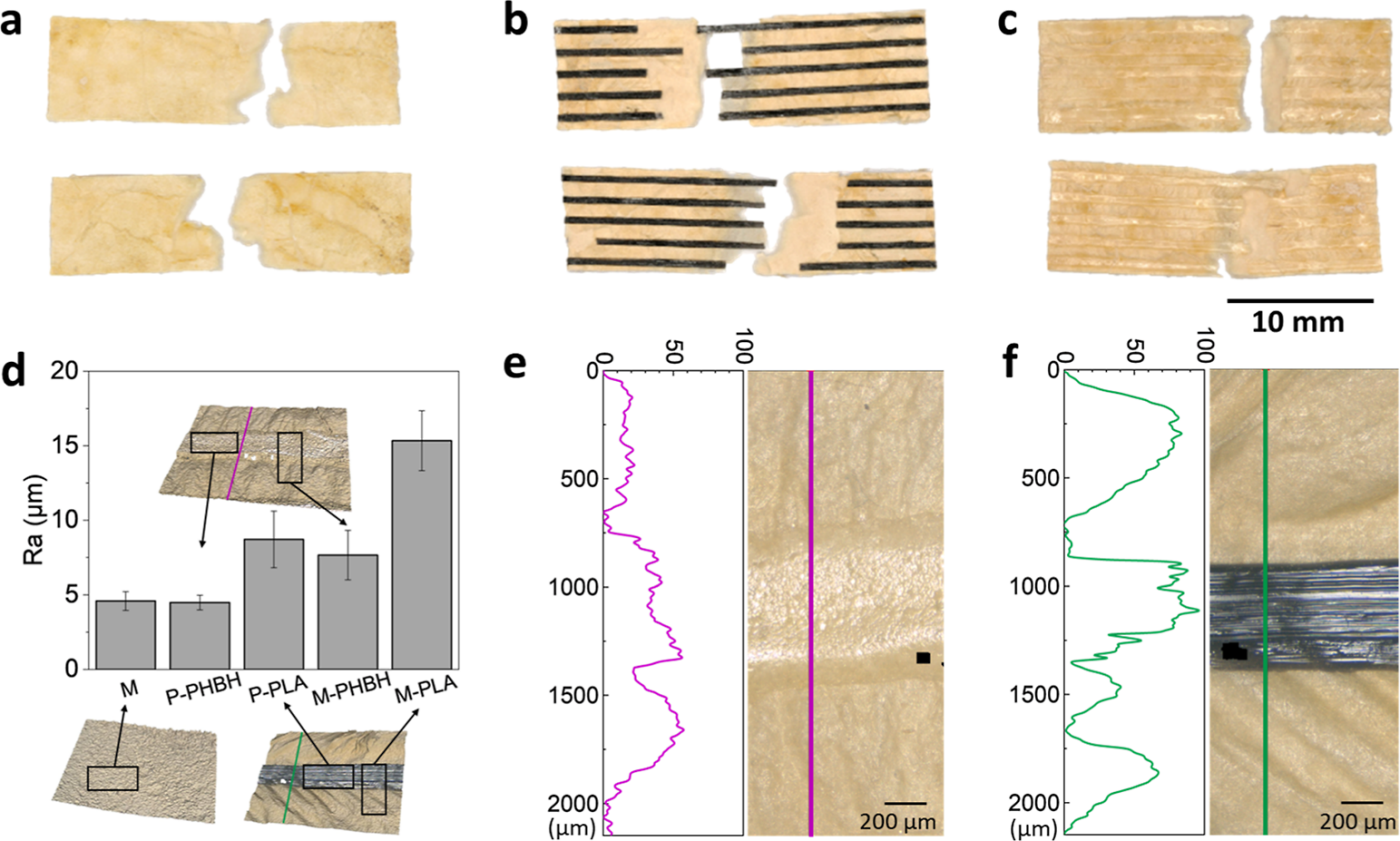
Light microscopy images of fractured (a) cold-pressed mycelium, (b) PLA printed on cold-pressed mycelium, and (c) PHBH printed on cold-pressed mycelium mats. Images were taken after tensile testing. (d) Roughness of cold-pressed pure fungal mycelium mats (M), printed PHBH and PLA filaments without (P-PHBH and P-PLA), or with (M-PHBH and M-PLA) fungal mycelium mats. Profile plots of representative filaments of (e) M-PHBH and (f) M-PLA on fungal mycelium mats.

In Figure 3a–c, the fracture of two specimens from each sample group can be seen. In Figure 3a, the cold-pressed pure fungal mycelium mat underwent a tensile test. The fracture is limited to a certain area and has a jagged shape. In Figure 3b, it can be seen that PLA breaks at a different point than the mycelium. In addition, a translucent mycelium layer can be seen between the PLA strands, which presumably corresponds to that additional layer in the SEM images. Also, in the case of PHBH printed on a fungal mycelium mat (Figure 3c), the location of the fracture of PHBH lines and mycelium does deviate from each other.

The roughness measurements (Figure 3d) show similar roughness (Ra) values for pure fungal mycelium mats (M: 4.59 ± 0.63 μm) and PHBH filaments (PHBH: 4.49 ± 0.49 μm). The roughness for printed PLA is around 2 times higher, which is 8.71 ± 1.90 μm. When taking both mycelium and polymer into consideration, the roughness is around two times higher but the difference keeps the same folder for M-PLA (15.34 ± 2.01 μm) and M-PHBH (7.66 ± 1.66 μm). The profile plots of M-PHBH (Figure 3e) and M-PLA (Figure 3f) indicate that the printed biopolymers have different impacts on changing the surface morphology of the fungal mycelium mat; while the difference in height between the PHBH and the substrate is less apparent in the profile plot, PLA has easily recognizable peaks on the image due to its prominent morphology. Surface distortion due to scratches from the printhead also shows up as one or more peaks on the profile plot, especially in M-PLA specimens.

## 3 Discussion

We propose a novel yet simple method to improve and adapt the mechanical properties of fungal mycelium mats. Chemical, physical, and thermal treatments have been used so far to improve the mechanical properties of mycelium mats. In our approach, the mats derived from surface fermentation in the liquid state are improved by depositing defined patterns of different biopolymers on them by using 3D printing. To evaluate the impact and quality of the biopolymer deposition, we compare the results of tensile test, SEM imaging, and roughness measurements.

By comparing the stress–strain curves of pure polymers, pure fungal mycelium mats, and polymer-deposited mats, the polymers efficiently increase the Young’s moduli as well as the ultimate tensile strength of the fungal mycelium mats. The ultimate tensile strength of M-PLA is about 4.8 times higher than that of pure fungal mycelium mat and about 2.8 times higher than that of M-PHBH. The Young’s modulus of M-PLA exceeds that of pure mycelium by a factor of 30 and that of M-PHBH by a factor of 3.5. Hence, in terms of mechanical stability, M-PLA shows the best performance. However, in each group of 8 samples tested, the standard deviation of Young’s moduli accounted for more than 42% of the mean. This is partly due to the inhomogeneity of the fungal mycelium cultivation process, as shown by the uneven thicknesses in the SEM images. Hence, the standard deviation of the Young’s modulus of pure fungal mycelium is already 38%. The deviation is further compounded by the fact that the biopolymers deposited at the later step connect or deform the surface to varying degrees, further increasing the error. During the tensile tests, because of their nonuniform thickness, the constraints in the clamps may be different, leading to possible errors such as slippage of individual filaments during the tensile test. These errors can be avoided by increasing the size of the sample and the density of the filament distribution. It is worth noting that in the SEM images, an extra thin but dense layer is visible, which may be the result of the deposition of components of the culture medium left on the surface of the fungal mycelium mat or a layer of densified mycelium. This extra layer could hinder the optimal adhesion of the filament to the mycelial mat. This can also be seen in Figure 3, which shows that the filament appears to adhere well to the extra layer that remains between the filament after breakage, while the extra layer detaches more easily from the rest of the mat. For both types of biopolymers, it can be seen that the filaments break at a location different from that of the fungal mycelium mat. This is consistent with the shape of the stress–strain curves, which show the initial loading of the filaments, and after the filaments break, the mycelium continues to be loaded. This leads to the assumption that the two materials act more as two independent entities. In the future, it could be interesting to evaluate the possibilities of improving the filament-mat adhesion by removing the extra layer, adjusting deposition parameters, functionalization, or additional steps of heat or optimized cold pressing. Nevertheless, combining the two material responses opens up new possibilities for smart applications.

Compared to the theoretical value calculated based on the Voigt model, both of the experimentally measured Young’s moduli are smaller. This deviation might come from the poor adhesion between the fungal mycelium mat and the biopolymer, making the result not simply the addition of two independent pure elastomers or pure viscosities. As shown in Figure 2, even for the same biopolymer, there are differences in the connection areas and contact angles with the substrate between different deposited lines. In addition to this, some wrinkled areas can be observed in the roughness measurements and the profile plots (Figure 3d–f), caused by the printhead scratching the surface of the fungal mycelium substrate during moving, which can also have an impact on the mechanical properties of the substrate.

The ultimate tensile strength and elastic modulus (0.65 ± 0.2 and 4.5 ± 1.7 MPa) of *F. fomentarius* mats used here are similar to those of *G. lucidum* obtained from potato dextrose-cellulose-based liquid medium (0.8 and 4 MPa)^60^ and mats of *Fomitella fraxinea* grown on solid substrate (1.40 ± 0.22 and 1.37 ± 0.20 MPa).^61^ After hot pressing at 120 °C, the ultimate tensile strength and elastic modulus of *F. fraxinea* were 3.99 ± 0.45 and 3.94 ± 0.46 MPa, respectively,^61^ with the ultimate tensile strength consistent with M-PLA (3.11 ± 0.5 MPa), while the elastic modulus is much higher (136.0 ± 57.7 MPa). Compared with the *Schizophyllum commune* sheets produced from fungal pellets from liquid shaken culture (ultimate tensile strength 5.0 ± 0.5 MPa and elastic modulus 468 ± 43 MPa),^62^ M-PLA has slightly lower ultimate tensile strength but much higher elasticity.

PHBH has a typical melting temperature of 145 °C at a heating rate of 10 °C/min, which is lower than the melting temperature of PLA (200–210 °C). To ensure a firm connection between the biopolymers and the fungal mycelium mat, a high extrusion temperature of 220 °C was applied to both polymers. As the deposited line always protrudes more or less from the surface of the fungal mycelium substrate, the leveling of print materials as well as the pattern and density of the print largely affects the surface roughness. At 220 °C, melted PHBH is assumed to be highly liquefied and has better leveling. Thus, we see bigger contact areas for PHBH, which might lead to better adhesion to the fungal mycelium mat, whereas PLA solidifies quickly after extrusion, leaving grooves where the nozzle edges have been scraped and therefore exhibiting greater roughness, which can be seen on the profile plot (Figure 3f). When the roughness is calculated by taking 50% each of the polymer and fungal mycelium mats, the overall roughness of M-PLA is also larger than that of M-PHBH.

The great advantage of materials derived from cultivated fungal mycelium is their compostability. Therefore, two compostable biopolymers were used in this study to improve the mechanical strength. While PLA has better mechanical properties and therefore improves the composite largely, it is only industrially compostable under precisely controlled conditions. In contrast, PHBH also improves the composite's performance but is home compostable.^58^ In this study, the same parameters for both polymers were used, which probably altered the final shape and adhesion of the filaments. Adjusting factors such as the extrusion temperature for PHBH might have a positive impact on the adhesion.

The big advantage of the MEX AM method is the easy adjustment of the line geometry. 2D auxetic structures^63^ could help to adjust the mechanical response and shift the stress peak along the elastic response of the fungal mycelium mat. In this way, unusual stress responses of natural materials could be mimicked. Other than that, applications similar to those of soft and flexible polymers are conceivable, such as smart textiles, flexible devices, and wearable electronics.^64,65^ Similar to cellulose-based foams, other areas of applications might be thermal insulation, separation, or porous substrates.

## 4 Conclusions

In this study, we present an innovative yet straightforward method to enhance and tailor the mechanical properties of fungal mycelium mats produced through liquid-state surface fermentation by utilizing MEX AM to deposit defined patterns of various biopolymers onto the mats. By using imaging methods, optical roughness measurement, and mechanical testing, the characterization of the composite material leads to the following conclusions:

- Load-path optimized compostable fungal mycelium-biopolymer composites can be manufactured via MEX AM.
- Industrially compostable PLA improves the mechanical properties to a greater extent compared with home-compostable PHBH.
- Adhesion and further interaction between the biopolymers and the mycelium mat can be optimized via printing parameters.

Looking forward, the production of fungal mycelium-biopolymer composites could improve stability, durability, design flexibility, and environmental responsiveness for further development of fungal mycelium materials in smart textiles, tissue engineering, and lightweight materials. To produce more stable products, the cultivation method and the pretreatment of the fungal mycelium mats before additive manufacturing have to be optimized.

## 5 Materials and Methods

### 5.1 Material Preparation

#### 5.1.1 Fungal Mycelium Mats Preparation

*F. fomentarius* fungal mycelium mats were cultivated with a liquid-state surface fermentation. The cultivation method was recently described in detail in Henning et al.,^66^ and here is the brief summary. After isolating from the main strain, fungal mycelium was first harvested from a 10–14 day-old agar plate culture with a sterilized scalpel. 1 L of complete medium was supplemented with 1 mL of 50 mg/mL ampicillin sodium salt and 1 mL of 50 mg/mL streptomycin sulfate to mitigate bacterial contamination risks. The isolated fungal mycelium was then inoculated into the complete medium. This mixture was homogenized using a sterile blender (300 W, Siemens AG, Germany) and transferred to a sterilized polypropylene container. The container was closed to prevent desiccation and external contamination. The subsequent incubation was carried out in the absence of light at a temperature range of 25–27 °C for 18–20 days. Postincubation, mature fungal mycelium mats were carefully taken out from the containers and placed on baking paper. Drying was performed in an oven at 50 °C for 2 days, with further deactivation of fungal mycelium at 70 °C for 3 h. In addition, to further improve the surface flatness of the fungal mycelium mats, they were cold pressed prior to additive manufacturing at 10 MPa for 10 min with a two-column electrohydraulic lab press (P/O/WEBER, PW 40E Lab Press).

#### 5.1.2 Material Extrusion Additive Manufacturing

Herein, we focus on polymer-based material to produce the tensile test specimens with MEX AM (also called fused filament fabrication).^67^ A Prusa i3MK3S+ (Prusa Research a.s., Prague, Czech Republic) with a 0.2 mm diameter nozzle and a Direct Drive System was used at an extrusion temperature of 220 °C. Further printing parameters can be found in Table S1. Printing parameters were defined in the open-source slicer software Ultimaker Cura.

#### 5.1.3 Material Extrusion Manufactured Pure Biopolymer Tensile Specimens

The biopolymer filament PLA was purchased from Filafarm (Filafarm GmbH & Co. KG, Germany) and used as received.^68^ PHBH filament was supplied by 3DK Trading GmbH (Berlin, Germany) and is claimed to be slightly modified from Kaneka (KANEKA Belgium NV, Belgium).^58^

The geometry of the tensile specimens was designed to fit into the ZwickRoell Z2.5 testing machine and determined according to the ASTM D3039 (Standard Test Method for Tensile Properties),^69^ which is 180 mm × 15 mm × 0.5 mm (length × width × thickness). A length of 120 mm is available for testing and 30 mm on each side for the tabs (Figure S2a,b). The height of the sample geometry was set to 0.5 mm. The single layer height of the printed specimens is 0.1 mm and the layer width is 0.2 mm. With a total height of 0.5 mm and a width of 15 mm for the specimen geometry, this results in 5 layers with 75 print paths each (Figure S2c).

The print parameters are provided in Table S1. The pure polymer specimens were printed with almost the same parameters as those of the polymer-mycelium specimens. Deviating print settings are noted in Table S1 after a slash (/). The first value refers to the pure biopolymer specimen, and the second to the biopolymer-mycelium specimen. Eight specimens were thus additively manufactured using the Prusa i3MK3S+ for each biopolymer, namely, PHBH and PLA.

#### 5.1.4 Material Extrusion Manufacturing of Biopolymer on Fungal Mycelium Mats

**Figure 4:**
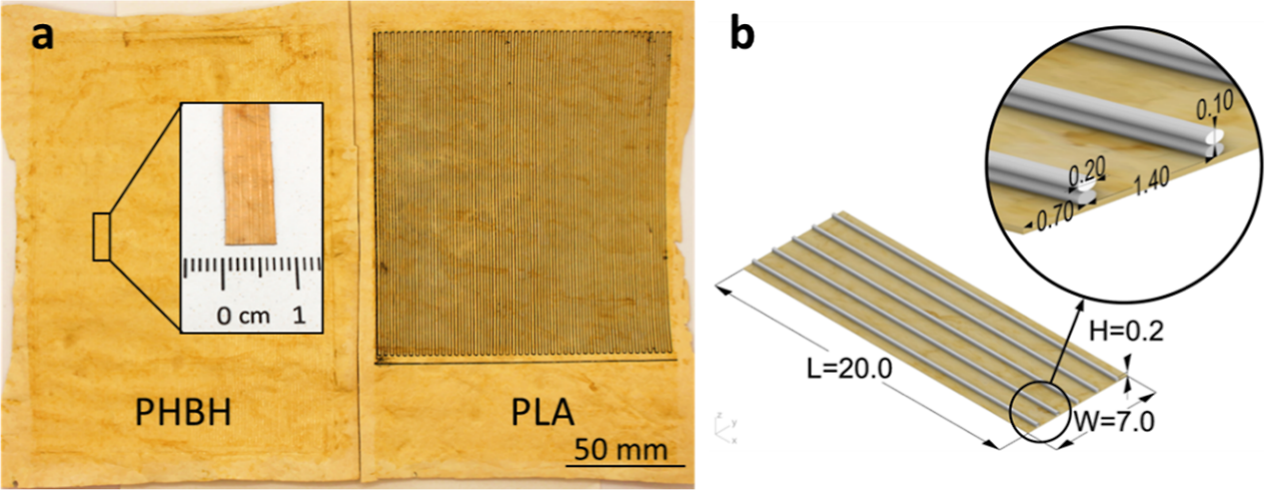
(a) Left: mycelium mat is printed with PHBH; right: mycelium mat is printed with PLA. (b) Schematic and detailed view of the test specimens, polymer printed on mycelium, unit in mm.

The specimens were designed to fit into the Kammrath & Weiss mechanical tester (Kammrath & Weiss GmbH, Dortmund, Germany) used for the tensile tests. Figure 4 shows the dimensions of the specimens. On the mycelium mat with dimensions *L* = 20 mm and *W* = 7 mm, there are five polymer lines, each 0.2 mm wide and 0.2 mm high. Each polymer line consists of two stacked print paths, each 0.1 mm high and 0.2 mm wide. The distance from the center of one path to the center of the next is 1.4 mm. For the production of the final samples, a mycelium mat that was approximately 200 mm long and 150 mm wide was attached to the Prusa i3MK3S+ printing bed with masking tape. The sliced pattern was subsequently printed with either PHBH or PLA onto the mycelium mat. The finished fungal mycelium mats were then cut with scissors to the sample dimensions.

### 5.2 Tensile Experiments

#### 5.2.1 Tensile Tests on Material Extrusion-Based Additively Manufactured Biopolymers

Uniaxial tensile tests were conducted to investigate the behavior of pure PHBH and PLA biopolymer specimens after MEX AM. The testing machine ZwickRoell Z2.5 (ZwickRoell GmbH & Co. KG, Germany) was used for all test series at a test speed of 1 mm/min (displacement control). The test automatically terminated when the specimens broke or when the minimum load was less than 20% of the maximum load.

Eight specimens of pure PHBH and PLA were tested to obtain the force–displacement relationship. The standard 2.5 kN load cell and the machine displacement were used as output signals. To calculate the stress (σ) and strain (ε) from each force–displacement curve, the dimensions of the specimen were measured by calipers. Mean stress–strain curves were calculated with a 95% confidence interval.

The Young's modulus *E* is determined according to the standard ASTM D3039^69^
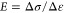
1in which the difference in tensile stress Δσ and the difference in strain Δε are determined between 0.001 and 0.003 strain.

#### 5.2.2 Tensile Tests on Biopolymer-Fungal Mycelium Composite

For conducting tensile testing, rectangular samples measuring 7 × 20 mm (Figure S3) were prepared and subjected to loading using a Kammrath & Weiss mechanical tester (Kammrath & Weiss GmbH, Dortmund, Germany). Force and displacement were measured using the built-in 500 N load cell, with an accuracy of 0.5 N, and the built-in displacement gauge with a range of ±6000 μm and an accuracy of 0.05 μm. These tests were conducted under displacement control mode at a speed of 20 μm/s.

To calculate stress and strain from the recorded force–displacement curves, the dimensions of each specimen were measured by using calipers. The mean curve, derived from an average of 8 experiments per composite variation, was calculated, along with a 95% confidence interval. And the Young's modulus *E* is determined according to the standard ASTM D3039.^69^

### 5.3 Imaging

#### 5.3.1 Digital Light Microscopy

After tensile testing, the fractured samples were imaged by Keyence VHX-7000 (Keyence Deutschland GmbH, Neu-Isenburg, Germany) in the stitching mode.

#### 5.3.2 Scanning Electron Microscopy

SEM was carried out in Quanta 400 ESEM (FEI, USA) by detecting backscattered electrons, and beforehand, the samples were sputtered for 15 s at 30 mA with gold in a sputter system (Sputter coater 108auto, Cressington, Germany).

#### 5.3.3 Roughness

The surface roughness (Ra) was measured by recording 3D pictures of the surface at 5-fold magnification using the Alicona infinite focus system (Alicona Imaging GmbH, Raaba/Graz, Austria). The result was analyzed in the 3D Image Viewer- Measure Suite 5.3.1 (Alicona GmbH). The mean surface roughness (Ra) along zigzag lines, with a total length of 4 mm, was calculated based on the obtained 3D images. Three specimens of pure mycelium mat and 5 specimens of each printed polymer were tested.

### 5.4 Prediction of the Elastic Properties of the Laminae with the Rule of Mixture

The prediction of the elastic constants of the two-phase material is well-described in the literature on composite materials (see, e.g., Altenbach et al.^70^ and Öchsner^71^). Herein, we determine the effective elastic properties in the longitudinal direction of the biopolymer-mycelium composite with parallel arrangement of the fibers and matrix, i.e., Voigt model, where the unidirectional composite is loaded with a tensile force parallel to the printed material. By assuming that the fibers (here: biopolymer) and the matrix material (here: fungal mycelium mats) experience the same extension that all components can be characterized by the one-dimensional Hooke’s law, the rule of mixtures of a simplified one-dimensional mechanical model can be derived as
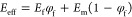
2where *E*_eff_ = effective Young’s modulus in the longitudinal direction, *E*_f_ = Young’s modulus of the fiber, *E*_m_ = Young’s modulus of the matrix, and φ_f_ = fiber volume fraction.

With the rule of mixtures presented in eq 2, we can determine the effective Young’s modulus of the laminae in the elastic range analytically. This is a very reasonable approach since the results shown in the previous section clearly depict a linear behavior in the initial part of the stress–strain diagrams (see Figure 1).

To determine the fiber volume fraction φ_*f*_, we

(a) determined the cross-section of the equivalent fibers (here: printed biopolymer lines) following the printing parameters as described in Table S1 and visualized in Figure 4b and
(b) measured the cross-section of each printed specimen prior to the testing and subsequently determined the mean value.

### 5.5 Statistical Methods

All experimental data were collected with at least three replicates. All numeric data are presented as the mean and standard deviation. Mean curves were calculated with a 95% confidence interval. Data were processed, and plots were generated with Pandas and Matplotlib libraries in Python.

## Data Availability

The original data are available from the corresponding author upon request.

## Supporting Information Available

SEM images of pure fungal mycelium mats, images of PHBH and PLA tensile specimen, a detailed schematic figure of pure biopolymer specimen, images of fungal mycelium mat before and after undergoing cold pressing, images of front, back, and side view of the tensile testing samples, and a table of printing parameters according to the slicer software (PDF)
