# Supplementary material for: Tailoring the Mechanical Properties of Fungal Mycelium Mats with Material Extrusion Additive Manufacturing of PHBH and PLA Biopolymers
Source: ACS Omega. 2024 Dec 3;9(50):49609–17. doi: 10.1021/acsomega.4c07661 (PMC11656364; doi:10.1021/acsomega.4c07661)
Supplement: Supplementary file 1 — ao4c07661_si_001.pdf [file ao4c07661_si_001.pdf]

Tailoring the Mechanical Properties of Fungal Mycelium Mats with Material Extrusion Additive Manufacturing of PHBH and PLA Biopolymers - supplementary materials

Huaiyou Chen[1‡*], Sophie Klemm[2‡], Antonia G. Dönitz[3], Yating Ou[3], Bertram Schmidt[4], Claudia Fleck[2], Ulla Simon[1], Christina Völlmecke[3]

[1]Technische Universität Berlin, Faculty III Process Sciences, Institute of Materials Science and Technology, Chair of Advanced Ceramic Materials, Berlin, 10623, Germany

[2]Technische Universität Berlin, Faculty III Process Sciences, Institute of Materials Science and Technology, Chair of Materials Science & Engineering/Fachgebiet Werkstofftechnik, Technische Universität Berlin, Str. des 17. Juni 135, 10623 Berlin, Germany

[3]Technische Universität Berlin, Faculty V Mechanical Engineering and Transport Systems, Institute of Mechanics, Chair of Stability and Failure of Functionally Optimized Structures, Berlin, 10623, Germany

[4]Technische Universität Berlin, Faculty III Process Sciences, Institute of Biotechnology, Chair of Applied and Molecular Microbiology, Berlin, 10623, Germany

[*]huaiyou.chen@tu-berlin.de

[‡]these authors contributed equally to this work

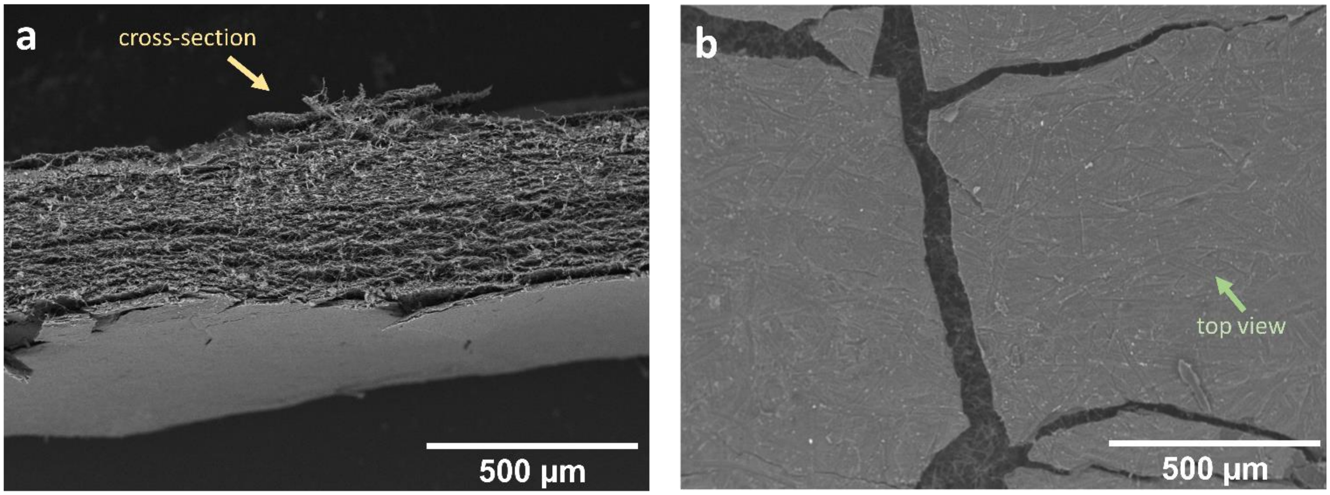

Figure S1 SEM images of **a**) the cross-section of a fungal mycelium mat and **b**) the top view showing a dense top layer of mycelium and more loosely compressed hyphae in the centre part. The magnification used is ×100.

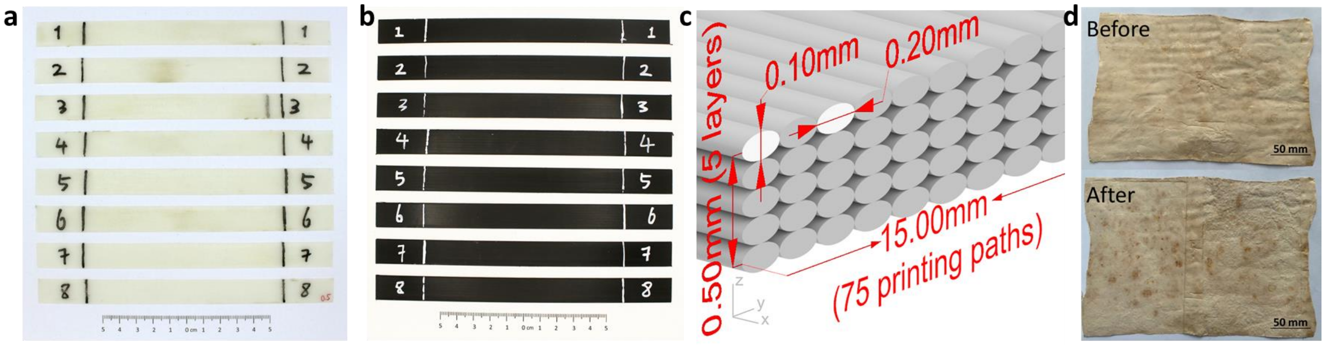

Figure S2 a) PLA tensile specimen, **b**) PHBH tensile specimen and **c**) detailed schematic figure of test specimen, number of layers and printing paths, **d**) fungal mycelium mat before and after undergoing cold pressing.

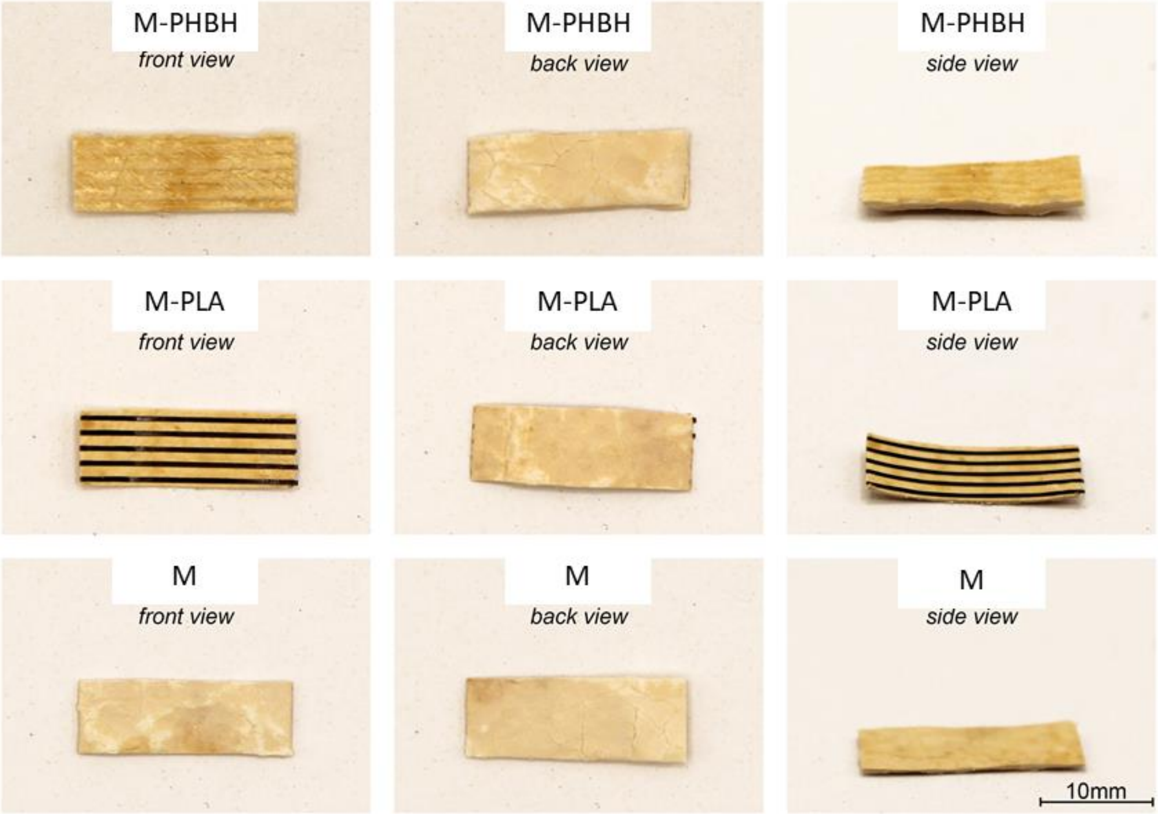

Figure S3 Front, back and side view of a representative specimen from each of the 3 test series

FFF 3D printer	
Printer	PRUSA i3 MK3S+
Nozzle Diameter	0.2 mm
**Quality**	
Layer Height	0.1 mm
Line Width	0.2 mm
**Walls**	
Wall Line Count	0
**Top/Bottom**	
Top/Bottom Layers	0
**Infill**	
Infill Density	100%
Infill Line Distance	0.2 mm/1.4 mm
Infill Pattern	Lines
Connect Infill Lines	off/on
Infill Line Directions	[90]
Infill Wipe Distance	1.0 mm
**Material**	
Printing Temperature	220°C
Build Plate Temperature	80.0°C
Flow	100%
**Speed**	
Print Speed	20.0mm/s
Travel Speed	100.0mm/s
Enable Jerk Control	on
**Travel**	
Enable Retraction	on
Retraction Minimum Travel	30.0 mm
**Cooling**	
Enable Print Cooling	off
**Build Plate Adhesion**	
Build Plate Adhesion Type	Skirt

Table S1. Printing parameters according to the slicer Ultimaker Cura
